# Knowledge of danger signs of pregnancy and health-seeking action among pregnant women: a health facility-based cross-sectional study

**DOI:** 10.1097/MS9.0000000000000610

**Published:** 2023-04-11

**Authors:** Shumiye Shiferaw Gesese, Eleni Adimassu Mersha, Wondu Feyisa Balcha

**Affiliations:** a Department of Midwifery, College of Medicine and Health Sciences, Bahir Dar University, Bahir Dar, Ethiopia; b Department of Reproductive Health and Population Studies, College of Medicine and Health Sciences, Bahir Dar University, Bahir Dar, Ethiopia

**Keywords:** antenatal care, danger signs, health-seeking action, knowledge, obstetric complication, pregnancy

## Abstract

In many developing countries maternal morbidity and mortality are challenging issues. Increasing the knowledge of women about the danger signs of pregnancy is the first essential step to reduce the adverse outcomes of pregnancy and delay in deciding to seek obstetric care, which in turn improves early detection of obstetric complications. This study aimed to assess knowledge of danger signs of pregnancy and health-seeking action among pregnant women.

**Methods:**

A health facility-based cross-sectional study was conducted in public health facilities from 1 March 2017 to 30 April 2017 on 414 pregnant mothers. The data were collected by systematic random sampling technique, entered into a computer using Epi data 3.5, and analyzed using Statistical Package of Social Sciences version 20.0. Bivariate and multivariable logistic regression analyses were done to estimate the crude and adjusted odds ratio with a confidence interval of 95% and a *P* value of less than 0.05 considered statistically significant.

**Results:**

This study identified that 57.2% of pregnant women had good knowledge of the danger signs of pregnancy. Pregnant women who are found in the age group of 25–29 [adjusted odds ratio (AOR)=3.35, 95% CI=1.13–9.96], and ≥30 years (AOR=8.11, 95% CI=2.23–29.45), mothers who live in urban area (AOR=5.26, 95% CI=1.96–14.15), primary education (AOR=4.85, 95% CI=2.07–11.41), secondary and above educational level (AOR=6.90, 95% CI=3.28–14.49), employed mother (AOR=5.18, 95% CI=1.65–16.27), being multigravida (AOR=7.24, 95% CI=3.86–13.58), knows that danger signs of pregnancy may cause severe complications (AOR=9.94, 95% CI=5.23–18.93), knew what to do if they faced danger signs of pregnancy (AOR=3.37, 95% CI=1.14–9.93), knew when did they go to a health facility if they faced danger signs of pregnancy (AOR=3.97, 95% CI=1.67–9.47) and faced at least one danger signs of pregnancy in current pregnancy (AOR = 5.40, 95% CI=1.46–19.99) were significantly associated with knowledge of danger signs of pregnancy. The proportion of mothers who experienced danger signs of pregnancy was 27 (6.5%) and among them, 21 (77.8%) had an appropriate health-seeking action, which is visiting a health facility.

**Conclusion:**

In this study area, the knowledge of pregnant women about the danger signs of pregnancy was low, while the practice of the mothers in response to danger signs of pregnancy was encouraging. Therefore, it is needed to the empowerment of women by increasing access to get an education, especially for rural women.

## Introduction

Highlights

- Lack of knowledge about obstetric danger signs of pregnancy and delay in seeking care are the major factors, which contribute to maternal death.
- This study showed that 57.2% of pregnant women had good knowledge of the danger signs of pregnancy, and 58.7% of pregnant women identified vaginal bleeding as a major danger sign of pregnancy
- Pregnant women’s knowledge of the danger signs of pregnancy increases with increasing their educational level.
- Pregnant women who know the danger signs of pregnancy were more likely to know what to do if they could face a problem during pregnancy.
- In our study, 27 (65%) of the pregnant mothers experienced at least one danger sign of pregnancy and among them, 21 (77.8%) had an appropriate health-seeking action, which is visiting a health facility.

Every pregnant woman faces the risk of sudden, unpredictable complications that could end up in death or injury to herself or to her infant that are related to obstetric danger signs of pregnancy^1^. Globally, around fifteen percent of pregnancies develop a potentially life-threatening complication that calls for skilled care and some will require a major obstetrical intervention to survive^2^. Worldwide, around 80% of maternal deaths are due to complications of pregnancy and childbirth and the average maternal mortality rate is 216 and 12 per 100 000 live births in developing countries and developed countries, respectively^3^. The vast majority of these deaths occurred in low-resource settings and Sub-Saharan Africa alone accounted for roughly two-thirds (196 000) of maternal deaths^4^. Deaths due to complications of pregnancy and childbirth can be easily prevented^5^. Delay in seeking care is one of the key factors leading to maternal death, which can be associated with a lack of knowledge about obstetric danger signs of pregnancy^6^.

Obstetric danger signs of pregnancy include severe vaginal bleeding, swollen hands/face, blurred vision, severe headache, loss of foetal movement, abnormal body movement, high-grade fever, severe abdominal pain, epigastric pain, excessive vomiting, the difficulty of breathing, loss of consciousness and abnormal vaginal discharge^7^–^17^. The presence of at least one obstetric danger sign of pregnancy indicates the need for immediate care for the woman^18^. Having antenatal care (ANC) visits is one of the entry points to offer health information and inform women about the danger signs and symptoms for which immediate assistance should be sought from a healthcare provider^19^. In Ethiopia, the systematic and meta-analysis showed that the pooled prevalence of women’s knowledge about obstetric danger signs during pregnancy was 48%^20^, and it ranged from 7.9 to 58.8%^12^,^21^.

Studies conducted in different countries revealed maternal and husband education level, occupation, maternal age, residency, parity, gravidity, family size, family income, decision-making power, number of ANC follow-ups, the timing of starting ANC visits, place of delivery, sources of information about obstetric danger signs and distance to health facility were some of the predictors of mothers’ knowledge of obstetrics danger signs of pregnancy^7^,^9^,^11^,^12^,^15^–^17^,^22^. The new United Nations Sustainable Development Goals (SDG) have three main targets, which are likely to reduce the maternal mortality rate below 70 deaths per 100 000 live births, the number of neonatal mortalities to 12 per 1000 live births, and the number of the death rate among under-5-year children by 25 per 1000 live births^23^. To achieve this goal, improving knowledge of obstetric danger signs of pregnancy is needed. Having good knowledge of obstetric danger signs during pregnancy can increase women’s capacity to ensure timely skilled care and safe birth^24^. Lack of awareness about obstetric danger signs contributes to delays in seeking and receiving skilled care^18^, while early recognition of these danger signs is very important to avoid delay in the decision to seek healthcare^25^. Even though health-seeking action was poor in developing countries, more than three-fourths of women were having appropriate health-seeking action after recognizing the danger signs in their pregnancy^26^,^27^.

Health-seeking behaviour is affected by socio-cultural factors: such as maternal age, educational levels, health insurance, ANC services utilization, knowledge of the danger signs of pregnancy, attitudes, and beliefs^28^–^31^. Recognizing danger signs can reduce the first delay to seek healthcare which in turn reduces maternal morbidity and mortality. However, still, large numbers of women die from a wide range of complications in pregnancy, particularly in developing countries including Ethiopia^27^. Most of these life-threatening complications are preventable and avoidable^32^. If a woman and her family have been pre-informed of obstetric danger signs of pregnancy, she will seek appropriate medical advice and the problems identified easily before complications arise, which makes a difference between life and death. However, the lack of knowledge about the danger signs of pregnancy and its complications is one of the reasons for the low level of women recognizing and seeking proper emergency care.

## Methods

The Institutional Review Board of Bahir Dar University, College of Medicine and Health Sciences has approved this study. All patients who were subjects of this study complied with the ethical standards set out in the Declaration of Helsinki in 1964. This study was also submitted and registered at www.researchregistry.com with a unique identification number researchregistry8731:https://www.researchregistry.com/browse-the-registry#home/. This work has been reported in line with the STROCSS criteria^33^.

### Study design and period

A health facility-based cross-sectional study design was employed from 1 March 2017 to 30 April 2017 in public health facilities.

### Study setting

The study was conducted in the public health facilities of Bahir Dar city. Bahir Dar City is the capital city of the Amhara Region and located ~565 km northwest of Addis Ababa, the capital city of Ethiopia. The city has a total population of 518 193 of which 265 156 are females^34^. All public health facilities of the city provide ANC services and included in this study.

### Study population

The study included systematically selected consenting pregnant mothers who attended ANC in public health facilities.

### Inclusion criteria and exclusion criteria

Pregnant women who attended the ANC in public health facilities were included, while pregnant women who revisited the ANC unit during the study period were excluded.

### Sample size determination

The sample size was calculated using a single population proportion formula by considering the following assumptions: knowledge of obstetrics danger signs of pregnancy in the previous study was 57.5%^35^, Zα/2= critical value for normal distribution at 95% confidence level, which is equal to 1.96 (Z value of alpha=0.05) or 5% level of significance (α=0.05) and a 5% margin of error (ω =0.05). The sample size was adjusted by adding a 10% non-response rate and the final total sample size was 414.

### Sampling procedure and technique

This study was conducted in eight public health facilities. After considering all of the public health facilities of the city, the total sample size was proportionally allocated for each public health facility in the city based on their monthly ANC flow, and by using the following formula.


$$\mathrm{Sample}\;\mathrm{in}\;\mathrm{the}\;\mathrm{facilities}=\mathrm{total}\;\mathrm{sample}\;\left(n\right)\times \frac{\mathrm{population}\;\mathrm{in}\;\mathrm{the}\;\mathrm{health}\;\mathrm{facility}\;\left(\mathrm{Ni}\right)}{\mathrm{total}\;\mathrm{population}\;\left(N\right)}$$

Then eligible pregnant women in each facility were selected by using systematic random sampling techniques based on their monthly ANC visits. The sampling interval or the K^th^ units was obtained by dividing the numbers of monthly pregnant women who attended ANC in the three public health facilities by the sample size of the study. The starting unit was selected by using the lottery method among the first k^th^ units in each facility.

### Study variables

#### Dependent variable

Knowledge of danger signs of pregnancy

#### Independent variables

Sociodemographic factors (maternal age, residency, marital status, religion, educational level, and occupation of mothers), and obstetric and other factors (gravidity, History of ANC visits in the previous pregnancy, complications of obstetric danger signs of pregnancy, what to do if danger signs of pregnancy develop when going to a health facility if they develop danger signs of pregnancy, faced danger signs in the current pregnancy, types of faced danger signs and sources of information for danger signs of pregnancy).

### Operational definitions

#### Danger signs

Are not actual obstetric complications, but symptoms and signs that are easily recognized by non-health professionals persons^11^,^36^.

#### Obstetric danger signs

Obstetric danger signs are unexpected obstetric signs that can lead to maternal health complications^16^.

#### Knowledge of danger signs of pregnancy

It was evaluated by the women’s answers to the danger signs of pregnancy-related questions. The obstetric danger signs of pregnancy questions were assessed by ‘+1’ for the correct answer and ‘0’ for the incorrect answer. The score for each mother was summed and categorized. The woman was considered to have good knowledge if she correctly answered greater than or equal to the mean score of the total knowledge assessing questions^11^.

#### Health-seeking actions

It refers to the health-seeking action a woman took after recognizing the danger signs during pregnancy. It was categorized as appropriate which is visiting a health facility or inappropriate which includes taking no action, consulting a friend/relative, self-medication, or consulting a traditional birth attendant/traditional healer^27^.

#### Antenatal care

Care provided by skilled healthcare professionals to pregnant women to ensure the best health outcomes for both the mother and baby during pregnancy^37^.

### Data collection tools and procedures

The questionnaire was developed by authors after reviewing different kinds of literature on the topic and validated by professional experts. Data were collected using structured and pre-tested interviewer-administered questionnaires through face-to-face exit interviews, which consists of sociodemographic characteristics, obstetric information, and knowledge practice-related questions. The questionnaire was first prepared in English and then translated to Amharic (local language) and back to English again by a language expert to maintain consistency. The data were collected by eight BSc midwives and, and supervised by two MSc midwives.

### Data quality control

Data were collected by trained data collectors and pre-testing of the instrument was done before the actual data collection. The questionnaire was pre-tested on 5%^21^ of pregnant women who attended ANC at a nearby health centre, which is not included in this study. Data collectors and supervisors were trained for two days by the investigator. After necessary modifications and correction was done to standardize and ensure its reliability and validity additional adjustments were made based on the results of the pre-test and daily supervision was done.

### Data processing and analysis

The data were entered into Epi data 3.5, edited and cleaned for inconsistencies, missing values, and outliers, then exported to the statistical package of the social science 20.0 version for analysis. During analysis, all explanatory variables which have a significant association in bivariate analysis with a *P* value less than 0.20 were entered into a multivariable logistic regression model to get an adjusted odds ratio (AOR), and those variables with 95% of CI and a *P* value of less than 0.05 was considered as statistical significance with knowledge of danger signs of pregnancy. The multicollinearity test was done using the variance inflation factor and no collinearity exists between the independent variables. The model goodness of the test was checked by Hosmer–Lemeshow goodness of the fit test and its *P* value was 0.776. Frequency tables, figures, and descriptive summaries were used to describe the study variables.

## Results

### Sociodemographic characteristics of the mothers

A total of 414 mothers participated in the study with a response rate of 100%. The mean age of the mothers was 25 years with (±SD=4.5) and 162 (39.1%) were found in the age group of 20–25 years. Of the mothers, 368 (88.9%) live in the urban area and 355 85.8%) are followers of the orthodox Christianity religion. About half (51.0%) of the mothers had secondary and above educational level and 255 (61.6%) are a housewife (Table 1).

**Table 1:** Sociodemographic characteristics of pregnant women who attended ANC in public health facilities, (*n*=414).

Variables	*N* (%)
Maternal age in years	
15–19	38 (9.2)
20–25	162 (39.1)
26–30	160 (38.6)
≥31	54 (13.1)
Residence	
Rural	46 (11.1)
Urban	368 (88.9)
Religion	
Orthodox	355 (85.8)
Muslim	51 (12.3)
Protestant	8 (1.9)
Marital status	
Married	403 (973)
Others^a^	11 (2.7)
Educational level	
No formal education	113 (27.3)
Primary education	90 (21.7)
Secondary and above	211 (51.0)
Occupational status	
Housewife	255 (61.6)
Merchants/self-employed	98 (23.7)
Employed (GO/NGO)	61 (14.7)

ANC, antenatal care.

a Single, divorced, and widowed.

### Knowledge of obstetrics danger signs of pregnancy

**Figure 1:**
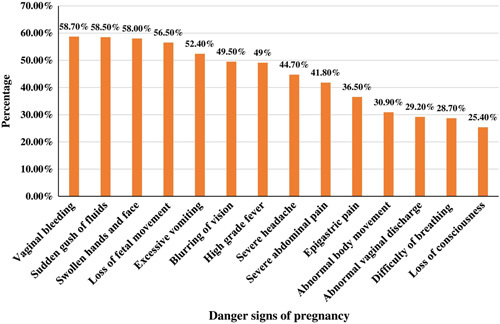
Danger signs of pregnancy among pregnant women who attended ANC in the public health facilities, (*n*=414). ANC, antenatal care.

**Figure 2:**
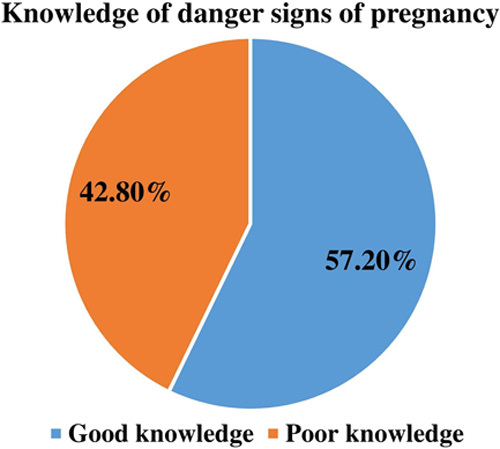
Knowledge of danger signs of pregnancy among pregnant women who attended ANC in the public health facilities, (*n*=414). ANC, antenatal care.

**Figure 3:**
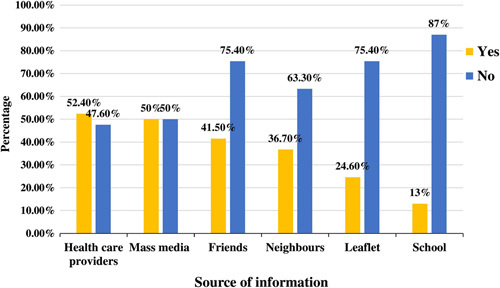
Source of information about danger signs of pregnancy among pregnant women who attended ANC in the public health facilities, (*n*=414). ANC, antenatal care.

More than half of the mothers, 243 (58.7%), 242 (58.5%), 240 (58.0%), 234 (56.5%), 217 (52.4%), 205 (49.5%), 203 (49.0%), and 185 (44.7%) of mothers responded vaginal bleeding, a sudden gush of fluids, swollen hands and face, loss of foetal movement, excessive vomiting, blurring of vision, high-grade fever, and severe headache as major obstetric danger signs of pregnancy respectively. The less identified danger signs of pregnancy are epigastric movement (36.50%), abnormal body movement (30.90%), abnormal vaginal discharge (29.20%), the difficulty of breathing (28.70%), and loss of consciousness (25.40%) (Fig. 1). Based on the predetermined criteria, this study identified that 237 (57.2%) [95% CI=52.2–61.8%] mothers had good knowledge of obstetric danger signs of pregnancy (Fig. 2). Of the mothers, 217 (52.4%), and 207 (50.0%) got information about the danger signs of pregnancy from health facilities and mass media respectively (Fig. 3).

### Obstetric characteristics of the mothers

In this study, 220 (531%) of the mothers are primigravida and among multigravida mothers, 158 (81.4%) had a history of ANC visits in their previous pregnancy. Among multigravida mothers and those who had a history of ANC visits 141 (89.2%) had good knowledge of obstetric danger signs of pregnancy. While among multigravida mothers and those who had no history of ANC visits more than half (52.8%) had poor knowledge of obstetric danger signs of pregnancy. Additionally, simple regression analysis indicates that mothers who had a history of previous ANC visits were 9.27 times more likely to have good knowledge of obstetric danger signs of pregnancy at a *P* value of less than 0.05 [AOR=9.27, 95% CI=4.06–21.16). In our study, 259 (62.6%) of mothers stated that pregnant women might die from obstetric danger signs of pregnancy or its complications, and 346 (83.6%) responded that pregnant women must have to go to a health facility if they faced any danger signs of pregnancy. Of the mothers, 317 (76.6%) said that she has gone to a health facility immediately if she faces any danger signs of pregnancy, while 30 (7.2%) said to wait until the appointment day. In our study, the proportion of mothers who experienced at least one danger sign of pregnancy was 27 (6.5%) and among them, 21 (77.8%) had an appropriate health-seeking action, which is visiting a health facility. While 6 (22.2%) had an inappropriate health-seeking action like taking no action, consulting a friend/relative, self-medication, or consulting a traditional birth attendant/traditional healer (Table 2).

**Table 2:** Obstetric characteristics of pregnant women who attended ANC in public health facilities, (*n*=414).

Variables	*N* (%)
Gravidity	
Primigravida	220 (53.1)
Multigravida	177 (42.8)
Grand multi gravida	17 (4.1)
History of ANC visit in a previous pregnancy (*n*=194)	
Yes	158 (81.4)
No	36 (18.6)
Could obstetric danger signs of pregnancy cause severe complications or maternal death	
Yes	259 (62.6)
I don’t know	155 (37.4)
What do you do, if you face danger signs of pregnancy?	
Go to a health facility	346 (83.6)
Consulting a traditional birth attendant or informing the family/friends	68 (16.4)
If you face danger signs of pregnancy, when did you go to a health facility?	
Immediately	317 (76.6)
When the condition becomes severe	67 (16.2)
Wait until the next appointment day	30 (7.2)
Did you face any danger signs of pregnancy during this pregnancy	
No	387 (93.5)
Yes	27 (6.5)
Which danger signs of pregnancy did you face in your current pregnancy (*n*=27) (multiple answers were possible)	
Excessive vomiting	14 (53.8)
Severe headache	12 (46.2)
Vaginal bleeding	8 (30.7)
Swelling of hand or face	7 (26.9)
Blurring of vision	4 (14.8)
Decreasing foetal movement	2 (7.7)
An action taken by pregnant women who faced at least one danger sign of pregnancy (*n*=27) (multiple answers were possible)	
Appropriate	21 (77.8)
Inappropriate	6 (22.2)

ANC, antenatal care.

### Factors associated with knowledge of obstetric danger signs of pregnancy

In bivariate analysis maternal age, residency, educational level, occupation, gravidity, knowing that obstetric danger signs of pregnancy may cause severe complications/maternal death, knowing what to do if they faced danger signs of pregnancy, knowing when they go to a health facility if they faced danger signs of pregnancy, and faced at least one danger signs of pregnancy in their current pregnancy were significantly associated with the knowledge of danger signs of pregnancy at a *P* value of less than 0.2.

In a multivariable analysis maternal age group of 25–29 [AOR=3.35, 95% CI=1.13–9.96], and ≥30 years [AOR=8.11, 95% CI=2.23–29.45], mothers who live in urban area [AOR=5.26, 95% CI=1.96–14.15], primary education [AOR=4.85, 95% CI=2.07–11.41], secondary and above educational level [AOR=6.90, 95% CI=3.28–14.49], employed mother [AOR=5.18, 95% CI=1.65–16.27], multigravida mother [AOR=7.24, 95% CI=3.86–13.58], know that obstetric danger signs of pregnancy may cause severe complications/maternal death [AOR=9.94, 95% CI=5.23–18.93], knew what to do if they faced danger signs of pregnancy [AOR=3.37, 95% CI=1.14–9.93], knew when did they go to a health facility if they faced danger signs of pregnancy [AOR=3.97, 95% CI=1.67–9.47] and faced at least one danger signs of pregnancy in their current pregnancy [AOR = 5.40, 95% CI=1.46–19.99] were significantly associated with knowledge of danger signs of pregnancy at a *P* value of less than 0.05 (Table 3).

**Table 3:** Logistic regression analysis for knowledge of danger signs of pregnancy among pregnant women who attended ANC in the public health facilities, (*n*=414).

	Knowledge of danger signs of pregnancy				
Variables	Good	Poor	COR (95% CI)	AOR (95% CI)	*P* value
Maternal age in years					
15–19	15	23	1	1	
20–24	86	76	1.73 (0.84–3.56)	2.66 (0.89–7.97)	0.080
25–29	98	62	2.42 (1.17–5.00)	3.35 (1.13–9.96)	0.029^a^
≥30	38	16	3.64 (1.52–8.73)	8.11 (2.23–29.45)	0.001^a^
Residence					
Rural	14	32	1	1	
Urban	223	145	3.51 (1.81–6.82)	5.26 (1.96–14.15)	0.001^a^
Educational level					
Had no formal education	36	77	1	1	
Primary education	53	37	3.06 (1.72–5.46)	4.85 (2.07–11.41)	0.001^a^
Secondary and above	148	63	5.02 (3.07–8.23)	6.90 (3.28–14.49)	0.001^a^
Occupation					
Housewife	137	118	1	1	
Merchant/self-employed	48	50	0.83 (0.52–1.32)	1.32 (0.66–2.66)	0.432
Government employed	52	9	4.98 (2.35–10.53)	5.18 (1.65–16.27)	0.005^a^
Gravidity					
Primigravida	79	141	1	1	
Multigravida	158	36	2.76 (1.52–4.50)	7.24 (3.86–13.58)	0.001^a^
Could obstetric danger signs of pregnancy cause severe complications/maternal death					
No	45	110	1	1	
Yes	192	67	7.01 (4.49–10.93)	9.94 (5.23–18.93)	0.001^a^
Health-seeking actions, if you face danger signs of pregnancy					
Consulting a TBA or informing the family/inappropriate	12	56	1	1	
Go to a health facility/appropriate	225	121	8.68 (4.48–16.81)	3.37 (1.14–9.93)	0.028^a^
If you face danger signs of pregnancy, when did you go to a health facility?					
When the problem becomes severe/the next appointment day	21	76	1	1	
Immediately	216	101	7.74 (4.52–13.25)	3.97 (1.67–9.47)	0.002^a^
Did you face any danger signs of pregnancy in your current pregnancy					
No	216	171	1	1	
Yes	21	6	2.77 (1.09–7.02)	5.40 (1.46–19.99)	0.012^a^

ANC, antenatal care; AOR, adjusted odds ratio, COR, crude odds ratio.

a Significant at a *P* value of less than 0.05.

## Discussion

Knowing the danger signs of pregnancy is essential for taking appropriate health-seeking actions, which is visiting a health facility for care if danger signs of pregnancy are faced. This study identified about 57.2% [95% CI=52.2–61.8%] of mothers had good knowledge of danger signs of pregnancy. This finding was in line with studies conducted in Ethiopia like Tsegedie district (58.8%)^12^, Dilla university referral hospital (58.0%)^38^, Gedo town (57.5%)^35^, East Gojjam zone (55.1%)^39^. It was also nearly similar to a study done in Ghana (55.1%)^31^. However, it was higher than the studies conducted in different parts of Ethiopia, which ranged from 7.9 to 46.7%^7^,^9^,^11^,^13^,^15^,^21^,^22^,^26^,^27^,^40^–^43^. The possible explanation for this discrepancy might be the study sitting. In our study around 89% of the women lives in urban and nearly 73% of them had at least a primary and above educational level, thus may contribute to the higher knowledge of the danger sign of pregnancy. This could be expected that living in an urbanized area and being educated will increase the chances of having information related to health.

It was also higher than studies conducted in Tanzania (25.2%)^44^, Nigeria (24.9%)^45^, Egypt (26%)^28^, and India (29.3%)^36^. This difference might be due to the sociodemographic and cultural differences of the study participants. While the knowledge of danger signs of pregnancy in our study was lower than in studies conducted in Cameroon (73.3%)^16^ and Nepal (66.0%)^46^. The possible reason for this discrepancy might be the study participants, the study in Cameroon includes immediate postpartum women, while our study includes pregnant women. The immediate postpartum mothers could have a high chance of getting information about the danger signs of pregnancy, labour delivery, and the postpartum period when they are in labour or before the onset of labour.

This study revealed that vaginal bleeding (78.7%), a sudden gush of fluids (58.5%), swollen hand or face (58.0%), loss of foetal movement (56.5%), and excessive vomiting (52.4%) were the most frequently mentioned danger signs of pregnancy. While less than one-third of the pregnant women identified abnormal body movement (30.90%), abnormal vaginal discharge (29.20%), the difficulty of breathing (28.70%), and loss of consciousness (25.40%) as the danger signs of pregnancy. This finding is consistent with other studies conducted in different parts of Ethiopia and other countries^7^,^9^–^11^,^13^,^17^,^40^,^47^. This indicates that more than half of pregnant women may know at least one or more danger signs of pregnancy, while the remaining even may not be known one danger sign of pregnancy. This warrants giving information about the danger signs of pregnancy and its complications in the form of counselling or health education. Because every pregnant woman might be facing the risk of sudden, unpredictable danger signs of pregnancy and its complications.

Factors that positively affect the danger signs of pregnancy were maternal socio-demographic and obstetric characteristics. This study identified that the knowledge of danger signs of pregnancy increased with increasing maternal age. Mothers who are found in the age group of 25–29 and ≥30 years were 3.35 and 8.11 times more likely to have good knowledge of the danger signs of pregnancy, respectively. Similar findings were reported in other studies^12^,^15^,^16^,^31^,^36^,^48^,^49^. The possible explanation might be older women may have had experiences with obstetric danger signs in past pregnancies, thus may increase their level of knowledge of obstetric danger signs of pregnancy. This implies that young women in their first pregnancy may need more consideration when providing counselling and health education. Health education during antenatal care improves mothers’ knowledge about obstetric danger signs^10^. Living in an urban area increased the odds of having good knowledge of the danger signs of pregnancy by 5.26 times. Similar findings were reported in different studies^11^,^38^,^49^,^50^. This could be since urban residents have better access to health information and maternal health services as compared with rural residents. Additionally, this is further contributed by the high prevalence of lack of formal education and transportation.

Furthermore, the knowledge of the danger signs of pregnancy increased with increasing maternal educational level. Mothers who had primary, secondary, and above educational levels were 4.85 and 6.90 times more likely to have good knowledge of the danger signs of pregnancy. This finding is consistent with other studies conducted in different parts of Ethiopia and other countries^7^,^9^,^12^,^16^,^21^,^27^,^39^,^48^,^51^–^53^. Education gives the impression to play a positive role in increasing the knowledge of women about danger signs during pregnancy and its complications^54^. This might be because educated women have better access to reproductive health-related information than those who had no formal education.

Similarly, government-employed mothers were 5.18 times more likely to know the danger signs of pregnancy. This finding is in line with studies conducted in Goba district, Raya Kobo district Nekemte Town^7^,^27^,^28^,^35^,^41^. The possible reason might be that government-employed women are educated and have better access to health-related information than non-educated women. Additionally, the employed have their source of income, which may make them have to better access to health-related information.

Multigravida mothers were 7.24 times more likely to have good knowledge of the danger signs of pregnancy. Similar findings were reported in Ethiopia^38^–^40^ and Cameroon^16^. The possible explanation might be due to previous exposure to obstetric complications and getting information about pregnancy from healthcare providers. As we have seen in our study, 52.4% of the mothers obtained information about obstetric danger signs of pregnancy from health facilities and they have good knowledge of obstetric danger signs of pregnancy than those who got the information from other sources.

Mothers who know that danger signs of pregnancy may cause severe complications/maternal death were 9.94 times more likely to have good knowledge of obstetric danger signs of pregnancy. Knowing the obstetric danger signs of pregnancy and its complications may lead to timely access to appropriate emergency obstetric care^19^. Lack of knowledge of complications of danger signs of pregnancy has a direct relationship with the women’s educational level. Our study indicates that, among pregnant women who have no formal education only less than one-third (31.8%) of them, have good knowledge of the danger signs of pregnancy. While more than two third (66.8%) of educated pregnant women have good knowledge of the danger signs of pregnancy.

Knowing what to do if faced a problem during pregnancy (taking appropriate health-seeking actions, which is visiting a health facility) increased the knowledge of danger signs of pregnancy by 3.37 times relative to those who take inappropriate actions like doing nothing, consulting a friend/relative, self-care/treatment or consulting a traditional birth attendant/traditional healer. In our study, mothers who faced at least one danger sign of pregnancy were 5.40 times more likely to have had good knowledge of the danger signs of pregnancy. Furthermore, mothers who knew that they have to go to a health facility immediately if they faced the danger signs of pregnancy were 3.97 times more likely to have good knowledge of the danger signs of pregnancy.

In the current study, 27/414 (6.5%) faced at least one danger sign of pregnancy in their current pregnancy and among them, 21/27 [77.8%, 95% CI (63.0–92.6%)] mothers had an appropriate health-seeking behaviour. Similar findings were reported in studies conducted in Debre Berhan (86.3%) and Nekemte town (91.3%)^26^,^27^. However, the health-seeking behaviour in our study was higher than in studies conducted in Harar town (57.4%) and Tanzania (56%)^55^,^56^. The possible explanation for this discrepancy could be due to the differences in socioeconomic and health-seeking behaviours of the study participants. Knowing the danger signs of pregnancy and knowing what to do if faced danger signs of pregnancy are crucial steps to getting appropriate and timely obstetric care.

### Implications of the study

Early recognition of the danger signs of pregnancy helps to avoid delay in the decision to seek healthcare. Knowing what to do and when to go to a health facility if faced danger signs of pregnancy could make the pregnancy outcome favourable. The finding of this study indicates a need of continuing taking action to increase the mothers’ knowledge of danger signs of pregnancy and to improve their health-seeking action when they faced any danger signs of pregnancy. Based on this, further studies should be carried out in this study area at the community level by including the male partners.

### Limitations and strengths of the study

This study was restricted to women who visited public health facilities and not assessed the knowledge level of pregnant women who have no ANC visits or have ANC visits in private health facilities. Pregnancy is the responsibility of both parents, but our study did not assess the husbands’ knowledge level of danger signs of pregnancy. It was also not triangulated with a qualitative study. As a strength, this study was conducted by pregnant women, thus was helped to minimize the recall bias.

## Conclusion

In this study area, the knowledge of pregnant women about the danger signs of pregnancy was low. Maternal age of greater than 25 years, urban residency, having primary and secondary and above educational level, government employed, multigravida, knowing obstetric danger signs of pregnancy may cause severe complications/maternal death, knew that they have to go to a health facility if they faced problem during pregnancy, and knew that they have to go to a health facility immediately if they faced danger signs of pregnancy were the predictors of obstetric danger signs of pregnancy. Therefore, it is needed to strengthen the empowerment of women by increasing access to get an education, especially for rural girls because getting at least a primary education is an important element to increase their knowledge of their health. The healthcare providers working in the ANC unit should give appropriate information based on the standard about obstetric danger signs of pregnancy to increase the knowledge of pregnant women.

## Ethical approval

Ethical clearance was obtained from the Institutional Review Board of Bahir Dar University, College of Medicine and Health Sciences. A formal letter was also obtained from each public health facility of Bahir Dar city. The study was conducted according to the recommendations of the Declaration of Helsinki.

## Consent

Written informed consent was obtained from each study participant for those ages greater than 18 years and from parents/guardians for those ages less than 18 years. The purpose of the study was explained to each pregnant mother/guardians. All respondents were assured that the data would not have any negative consequences on any aspect of their life

## Source of funding

This study was financially supported by Bahir Dar University College of Medicine. The funder has no role in study design, data collection, analysis, interpretation, the decision to publish, or the preparation of the manuscript.

## Authors contribution

S.S.G. and E.A.M. were responsible for the conception of the research idea, study design, data collection, analysis and interpretation, and supervision. S.S.G. and W.F.B. participated in the data collection, entry, analysis, and manuscript write-up. All authors have read and approved the final manuscript.

## Conflicts of interest disclosure

All authors declare that they do not have any conflicts of interest.

## Research registration unique identifying number (UIN)

Not applicable.

## Availability of data

All related data have been presented within the manuscript. The data set supporting the conclusion of this article is available from the corresponding author upon reasonable request.

## Provenance and peer review

Not commissioned, externally peer-reviewed.
